# High-resolution studies of photo(electro)catalysts by electrochemical scanning probe microscopy

**DOI:** 10.1039/d6sc01523a

**Published:** 2026-05-04

**Authors:** Ziyuan Wang, Gaukhar Askarova, Tianyu Bo, Michael V. Mirkin

**Affiliations:** a Department of Chemistry and Biochemistry, Queens College-CUNY Flushing NY 11367 USA; b The Graduate Center of CUNY New York NY 10016 USA mmirkin@qc.cuny.edu

## Abstract

Photocatalytic processes are central to many energy and environmental applications; however, mechanistic understanding of these processes is incomplete. Traditional techniques provide bulk-averaged data, missing crucial activity variations between and within individual photocatalyst particles, which are challenging to probe due to their small size. Scanning electrochemical microscopy (SECM) and related techniques provide a unique spatially resolved perspective, enabling deeper insights into photocatalyst performance at the microscale and the nanoscale. In this article, we review applications of electrochemical scanning probe techniques in studies of particulate and two-dimensional photo(electro)catalysts and advancements in quantitative kinetic measurements and high-resolution mapping of CT and catalytic activity. The characterization of heterogeneous photocatalysts and co-catalysts using the recently developed tunnelling mode of photo-SECM and the expected synergy between amperometric and potentiometric nanoelectrochemical techniques are also discussed.

## Abstract 2

Scanning electrochemical microscopy and related techniques provide a unique spatially resolved perspective, enabling deeper insights into photocatalyst performance at the nanoscale.

## Graphical abstract

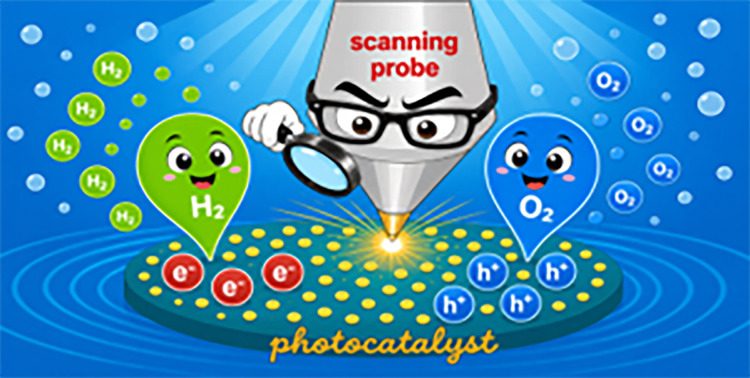

Since the first report of TiO_2_ as a light harvesting material,^1^ there has been significant interest in exploring the utility of various semiconductors and cocatalysts for water splitting and other photo(electro)catalytic processes.^2,3^ Many technologically important systems rely on particulate, two-dimensional (2D), and faceted semiconductor photocatalysts.^4–6^ Detailed knowledge of charge-transfer (CT) mechanisms on the nanoscale is required to design and optimize such systems. Photo(electro)catalysts and cocatalysts are known to be kinetically heterogeneous with different active sites (*e.g.*, structural defects and facet edges) contributing to the overall activity.^7,8^ Mapping reaction rates at the active sites of photo(electro)catalysts with a nanometer-scale spatial resolution^9^ and characterizing the kinetic heterogeneity of photocatalyst populations^10^ are crucially important for understanding and improving their activity. Recent progress in photo-scanning electrochemical microscopy (photo-SECM) and related techniques, including a significant increase in their spatial resolution, rendered these methods useful for nanoscale characterization of semiconductors and co-catalysts.^11–15^

## 1 Fundamentals of photo-SECM and related techniques

### 1.1 Illumination modes

**Fig. 1:**
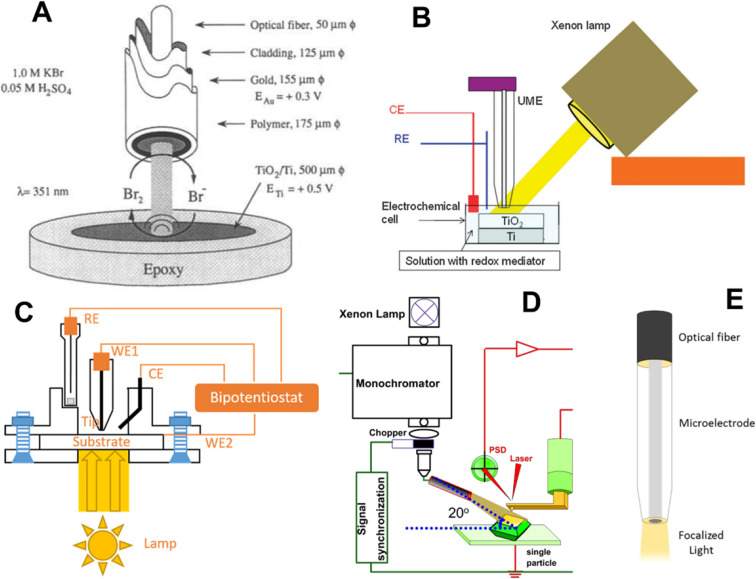
Different approaches to sample illumination employed in electrochemical scanning probe microscopy (SPM). (A) Schematic representation of a ring-type metal coated optical fiber.^26^ Global side^21^ (B) and bottom^25^ (C) illumination in SECM. (D) Side illumination in surface photovoltage microscopy (SPVM).^47^ (E) Through-tip illumination: schematic diagram of the microelectrode used simultaneously for local illumination and as a sensing probe in photo-SECM.^37^ Reproduced from ref. 21, 25, 26, 37 and 47 with permission from Royal Society of Chemistry, American Chemical Society, Electrochemical Society, and Springer Nature, copyright 1995, 2012, 2017, 2019, and 2024.

Photoelectrochemical applications of SPMs require the integration of a light source to irradiate the sample surface. Several reported experimental setups vary in how light is introduced into the system and how photoelectrochemical signals are collected (Fig. 1). Common approaches include global illumination of the entire sample surface (either bottom or side illumination) with localized detection of photogenerated species by the scanning probe;^16–22^ localized illumination of the sample by a focused laser beam or an optical fiber, while monitoring the overall photocurrent;^23–25^ a fiber-based scanning probe coated with concentric metal and insulating layers, functioning as a ring-type photoelectrode;^26–36^ and through-tip illumination, in which the light is guided through the glass body of the tip electrode to the area of interest.^37–46^

Global side illumination is a straightforward approach whose limitations include the possibility of light being reflected, scattered, or blocked by the body of the scanning probe. These optical interferences are most significant at short tip–substrate separation distances required for high-resolution imaging. These effects can broaden and distort the actual light distribution on the substrate surface. The illumination from the bottom of the substrate is free from these problems and can enable direct and uniform light delivery to the photoactive interface; however, it is only suitable for transparent or ultrathin samples. Furthermore, global illumination of a photoactive material often results in substantial photocurrent generation across the entire surface, which can lead to the accumulation of reaction products and obscure localized photoelectrochemical behavior. The species generated in sample regions far from the tip can diffuse into the tip–substrate gap and change the measured current. Consequently, the current is not determined solely by the local reaction rate directly under the probe, which can affect the spatial resolution and quantitative accuracy of local photocurrent, flux, or kinetic measurements.

To overcome these limitations, spatially confined illumination—typically achieved through focused light sources or optical fibers—is preferable, as it minimizes undesired product flux from non-local regions and enhances the spatial resolution of photocurrent mapping. The Schuhmann group introduced a through-tip illumination approach using glass-sealed platinum ultramicroelectrodes with a 25 µm disk geometry, in which an optical fiber was coupled to the rear of the electrode.^37,38^ In this configuration, the SECM probe simultaneously serves as an electrochemical sensor and a conduit for localized light delivery, thereby reducing artifacts associated with light scattering, reflection, and shading by the probe body as well as the contributions from distant parts of the substrate.

This concept was later extended to attain higher spatial resolution with nanometer-sized tips.^39,42^ Such probes are fabricated similarly to nanotips employed for SECM experiments without illumination: typically, a metal microwire is sealed into a glass capillary using a laser puller and polished to expose a disk-shaped conductive surface. With an optical fiber coupled to the back side of the probe, the glass body serves as both an insulating sheath for the electrode and an optical pathway for light delivery, whereas the exposed metal disk functions as the electrochemical sensor. Although the diameter of the illuminated area may be significantly larger than that of the glass sheath, the spatial resolution in photo-SECM is primarily governed by the conductive tip radius rather than the size of the light spot.

The problems associated with through-tip illumination include the uncertainty in the size of the light spot, light intensity and distribution on the sample surface. (No modeling of light propagation through the nanotip has been reported to date.) Significant light losses are expected to occur due to diffuse reflection on the metal surface, scattering on the air/glass interface, and especially during light propagation through the conical portion of the capillary.^42^ Additional light losses may be caused by imperfect tip-fiber alignment. Since the diameter of the illuminated area on the substrate surface depends on the tip/substrate distance, quantitative comparison of photocurrents obtained at different locations requires careful control of the probe geometry and separation distance.

### 1.2 Operational modes of photo-SECM

**Fig. 2:**
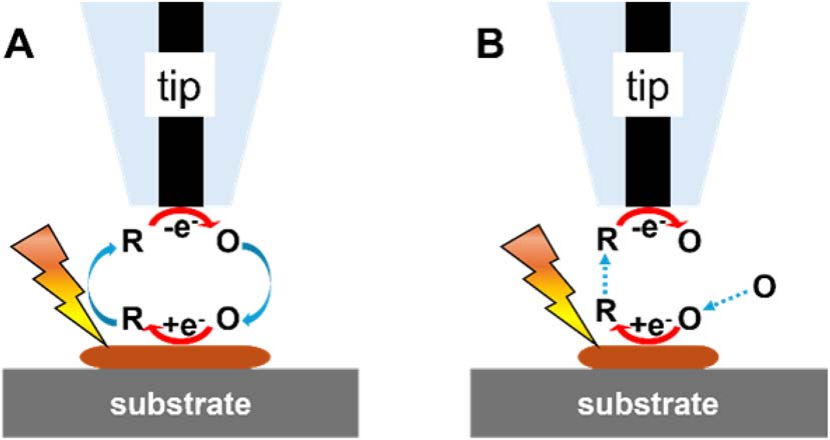
Feedback (A) and SG/TC (B) modes of photo-SECM operation.

The first photo-SECM study of photoelectrocatalysis dates back to 1997 when Fukushima and co-workers^18^ measured concentration changes of dissolved oxygen and H_2_O_2_ due to the photocatalytic reaction at the TiO_2_ surface coated with Pd. Afterwards, photo-SECM with micrometer-sized probes has been extensively employed for studying photoelectrochemical processes^31,32,36,48–51^ and screening photo(electro)catalytic properties of various materials.^25,52–55^ Two modes of the photo-SECM operation in such studies are illustrated in Fig. 2. In the feedback mode experiment (Fig. 2A), the electrolyte contains a redox mediator, and the tip potential (*E*_T_) is such that the mediator oxidation (or reduction) occurs on its surface at the diffusion-controlled rate.^11^ (To avoid possible interference of redox species added to the solution with photo(electro)chemical processes of interest, either the oxygen reduction reaction (ORR) or the hydrogen evolution reaction (HER) can be used as the source of tip current). When the separation distance (*d*) between the tip and either a biased or an unbiased substrate is small (*i.e.*, comparable to tip radius, *a*), the product of the mediator oxidation at the tip can diffuse to the illuminated substrate surface and get photoreduced (or oxidized) there. The regeneration of the mediator at the substrate leads to the increase in tip current (*i*_T_) with decreasing *d* (positive feedback; Fig. 2A). However, if the regeneration is slow, *i*_T_ decreases with decreasing *d* because the substrate surface blocks the diffusion of the mediator to the tip (negative feedback). In the substrate generation/tip collection mode (SG/TC; Fig. 2B), the flux of molecules is generated by a photo(electro)chemical reaction at the substrate surface and collected at the SECM tip. In this mode, one can observe different photoelectrocatalytic reactions occurring at the substrate depending on the applied bias (*e.g.*, the photoelectrochemical oxygen evolution reaction (OER) at positive potentials and the HER at negative potentials).

**Fig. 3:**
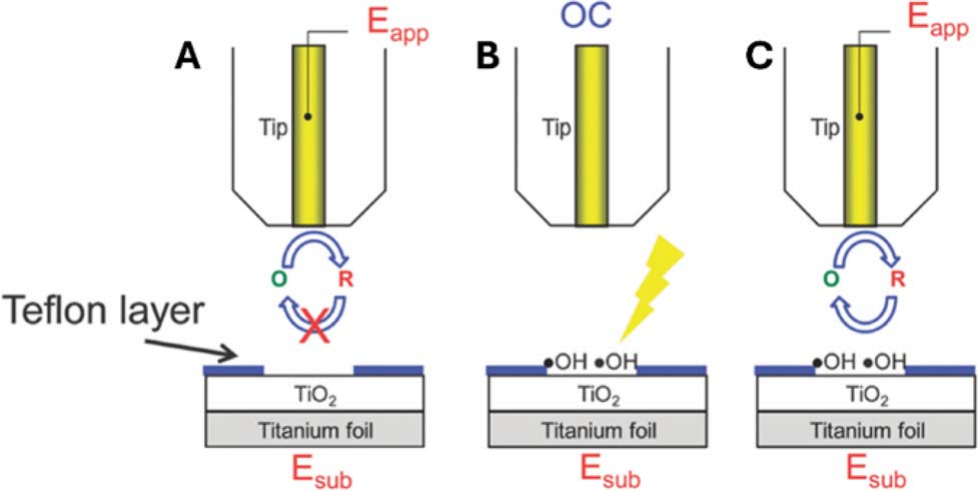
Photo-SI-SECM operation scheme for the reduction of photogenerated ˙OH_(ads)_ on TiO_2_. (A) No ˙OH_(ads)_ is produced in the dark; the substrate surface is inert. (B) Tip is at open circuit, and ˙OH_(ads)_ are generated on TiO_2_ through surface irradiation. (C) Light is switched off; tip-generated R species react with ˙OH_(ads)_. Adapted from ref. 21 with permission from Royal Society of Chemistry, copyright 2012.

The Bard group^21^ introduced a new concept of surface interrogation SECM (SI-SECM) as an *in situ* technique for detecting and quantifying photogenerated species on surfaces, *e.g.*, hydroxyl radicals adsorbed on TiO_2_ (˙OH_(ads)_). This development opened new avenues for studying the reaction dynamics of intermediates of photochemical processes at semiconducting materials. In SI-SECM, the tip is brought close to the substrate and biased at a suitable potential to reduce the dissolved mediator species (O) at a diffusion-limited rate (Fig. 3). With no hydroxyl radicals generated in the dark, this process yields negative feedback (Fig. 3A). Then, the tip is set to open-circuit potential, and the substrate is irradiated to accumulate ˙OH_(ads)_ on its surface (Fig. 3B). After a certain time interval, the light is switched off, and a negative potential is reapplied to the tip to measure the current transient due to the chemical reaction between the R species and ˙OH_(ads)_ on the substrate surface that regenerates O (Fig. 3C). The analysis of tip current transients yields the adsorbate coverage. The Rodríguez-López group^56,57^ employed smaller (*a* = 240 nm) tips for photo-SI-SECM characterization of adsorbed intermediates on both pristine and ion-milled defective areas of n-doped (100) SrTiO_3_ photoelectrodes. The measured bimolecular reaction rates showed significant variations, indicating that surface modification impacts the reactivity of photogenerated reactive oxygen species. The same group also used this technique to quantify reactive oxygen species intermediates formed on hematite photoelectrodes.^58^ Photo-SI-SECM experiments require precise control of the accumulation time and illumination geometry to avoid artifacts that may be caused by nonuniform light distribution and local heating. Some of those issues may be associated with global illumination configurations, where probe shading, reflection, and scattering can make the local photon flux difficult to control, leading to nonuniform accumulation of surface intermediates. Localized (*e.g.*, through-tip) illumination may help reduce these effects in future implementations of photo-SI-SECM.

### 1.3 Photo-SECCM

**Fig. 4:**
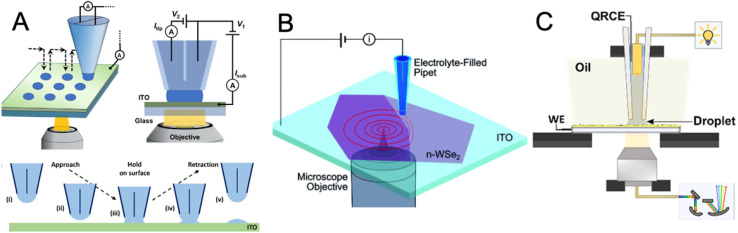
Schematic of photo-SECCM with hopping illustration. (A) Double-barrel pipette configuration with global bottom illumination. (B) Single-barrel pipette configuration with local laser bottom illumination – carrier generation-tip collection (CG-TC SECM). (C) Single-barrel pipette configuration with a top-down light path arrangement coupled with spatially resolved optical spectroscopy (SR-OS). Reproduced from ref. 61, 65, and 69 with permission from American Chemical Society and Royal Society of Chemistry, copyright 2024, 2021, and 2023.

An alternative approach to high-resolution mapping of photoinduced charge transfer and reactivity is based on photo-scanning electrochemical cell microscopy (photo-SECCM).^11,59^ SECCM uses a nano- or micropipette containing electrolyte and a quasi-reference/counter electrode to form a confined meniscus cell at the substrate surface (Fig. 4). SECCM probes may be configured as dual-barrel^59–61^ or single-barrel^62–70^ pipettes. Dual-barrel probes (Fig. 4A) are fabricated from theta (*θ*) capillaries and contain two electrolyte-filled channels, each housing a quasi-reference counter electrode (QRCE). This configuration provides an interchannel ionic current that serves as a reliable feedback signal for meniscus-contact detection and enables simultaneous ion-conductance and voltammetric measurements. However, the presence of two barrels increases the overall probe size, complicates meniscus wetting behavior, and requires more demanding fabrication procedures.

In contrast, single-barrel pipettes (Fig. 4B and C) contain only one electrolyte channel and a single internal QRCE that functions as the combined reference/counter electrode. This design offers a smaller, more symmetric meniscus that enhances spatial resolution and improves light access to the substrate, which is advantageous in photo-SECCM. In either configuration, the technique enables direct reading of the photocurrent flowing at the microscopic portion of the substrate surface under the meniscus. The lateral resolution is determined by the pipette meniscus size, allowing mapping of photoactivity at the micro- to sub-micrometer scale. The reported photo-SECCM studies have employed the hopping mode of scanning. In this regime, the probe performs a sequence of discrete approach–retract events arranged on a predefined grid in the *xy*-plane. Each approach/retract cycle corresponds to a single “hop,” with successive landing positions separated by a fixed lateral step size.

Although less versatile than photo-SECM (lower spatial resolution, no feedback or generation/collection experiments are possible), photo-SECCM can be useful for studying processes whose products are not electroactive and cannot be detected by the tip. Instead, photo-SECCM relies on the direct voltammetric (*e.g.*, cyclic voltammetry) or potentiometric (*e.g.*, open-circuit potential) interrogation of the photoreaction kinetics occurring at the substrate surface confined within the meniscus cell. It also offers the possibility to perform sub-micrometer scale experiments without demanding fabrication and characterization of well-shaped nanoelectrodes. Since photo-SECCM directly measures the current flowing on the portion of the substrate surface under the solution meniscus, the extraction of the local photoelectrocatalytic CT rate from it may be easier than the evaluation of the product flux from the SECM tip current. However, without SG/TC capability, separating the contributions of different CT processes simultaneously occurring on the photocatalyst surface is not straightforward. This technique also cannot be used to probe unbiased photocatalysts, where the overall process produces no net electric current.

An important potential source of errors and artifacts in SECCM experiments is the uncertainty of the contact area between the liquid meniscus and the substrate surface to which the measured current is directly proportional. Several approaches to ascertaining the contact area have been reported,^71^ including *in situ* optical monitoring^72^ and *ex situ* techniques based on SEM^73^ imaging of the contact footprint. However, this area, which depends on local wettability, can vary over the sample surface and change during the experiment at a given location. Moreover, the true surface area wetted by the droplet may be significantly increased by an extremely thin layer of water (nm or sub-nm thick) that forms on various surfaces^74^ and should be hard to detect and evaluate.

The choice of illumination geometry determines the efficiency and uniformity of light delivery to the SECCM meniscus and underlying substrate. Bottom illumination, implemented through a transparent substrate, is frequently used in studies of 2D semiconductors and ultrathin films; for instance, Hill and co-workers^62–66^ illuminated transition metal dichalcogenide (TMD) samples through the objective of an inverted microscope, enabling precise alignment between the optical focus and the SECCM probe (Fig. 4A and B). Side illumination is commonly used in photo-SECCM when the substrate is opaque or the surface morphology prevents uniform illumination through the substrate or along the probe axis;^67,68^ however, the precise photon flux at the confined meniscus is more difficult to quantify in this configuration. The Chen group^69,70^ implemented a probe-integrated illumination scheme in which an optical fiber was inserted into the micropipette to deliver light directly to the meniscus region (Fig. 4C). This configuration was designed to couple spatially resolved optical spectroscopy with SECCM, enabling one-to-one correlation between local photocatalytic responses and position-dependent properties such as absorption features and bandgap energies.

Photo-SECCM enables direct interrogation of photogenerated carrier transport in semiconductors, which has not yet been attained with photo-SECM. When tightly focused and spatially offset illumination is used, the SECCM signal reflects the transport of carriers from the excitation site to the confined meniscus cell. This forms the basis of the carrier generation–tip collection (CG-TC) mode, demonstrated by Hill and co-workers^65^ on n-WSe_2_, where SECCM visualized carrier recombination at individual defects such as step edges.

SECCM can also be used to interrogate photocatalytic behavior on rough or high-aspect-ratio architectures, where its confined meniscus enables localized measurements on complex surfaces. Makarova *et al.*^68^ applied photo-SECCM under UV illumination to TiO_2_ nanotube arrays and measured spatially resolved OER activity across both the tops and sidewalls of individual nanotubes, demonstrating the technique's ability to probe photochemical reactivity within structured, three-dimensional electrode architectures.

### 1.4 SPM methods for surface potential, interfacial fields, and charge transfer measurement

**Fig. 5:**
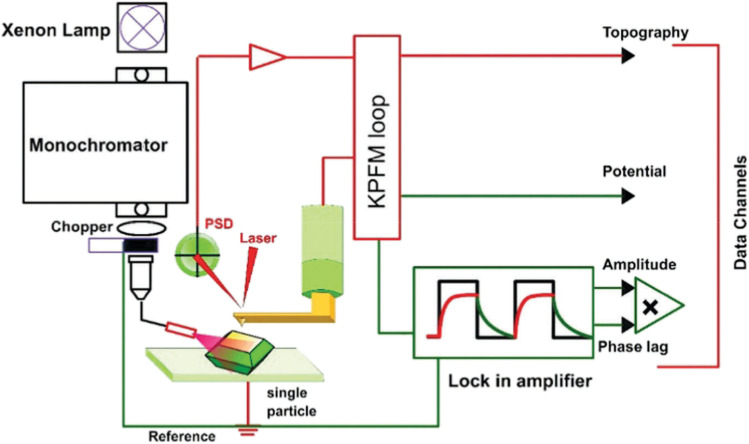
Schematic diagram of the setup used for spatially resolved SPV measurements, including the illumination module, the KPFM control loop, and the lock-in/data-acquisition electronics. Adapted from ref. 12 with permission from the Royal Society of Chemistry, copyright 2018.

Neither SECCM nor conventional SECM can directly quantify local surface potentials or the thermodynamic driving forces that govern interfacial CT processes. Several nanoscale scanning probe techniques can be combined with photo-SECM to build a more complete picture of charge generation, separation, transport, and interfacial CT in photo(electro)catalytic systems. Surface photovoltage microscopy (SPVM)^12,47,75^ implemented *via* Kelvin probe force microscopy (KPFM, Fig. 5) provides nanoscale maps of the surface potential changes under illumination relative to the dark state (SPV = *V*_light_ – *V*_dark_). Light generates electron–hole pairs and drives their redistribution among the bulk, surface states, and interfaces, which modifies band bending and produces a local quasi-Fermi-level splitting. SPVM, therefore, maps the spatial pattern of SPV (magnitude and sign), which directly reflects illumination-induced shifts in surface potential arising from local changes in band bending and photocarrier redistribution.

Unlike photo-SECM, which measures *operando* faradaic photocurrents in solution, SPVM is typically performed in air (or under a controlled atmosphere) and therefore cannot directly reflect solution-coupled charge transfer or the true (photo)electrocatalytic behavior under operating conditions. Instead, it reports illumination-induced potential changes with minimal interference from electrolyte screening, interfacial reactions, and mass transport. Taken together, SPVM and photo-SECM can provide complementary information by correlating intrinsic photovoltage with *operando* reactivity maps.

**Fig. 6:**
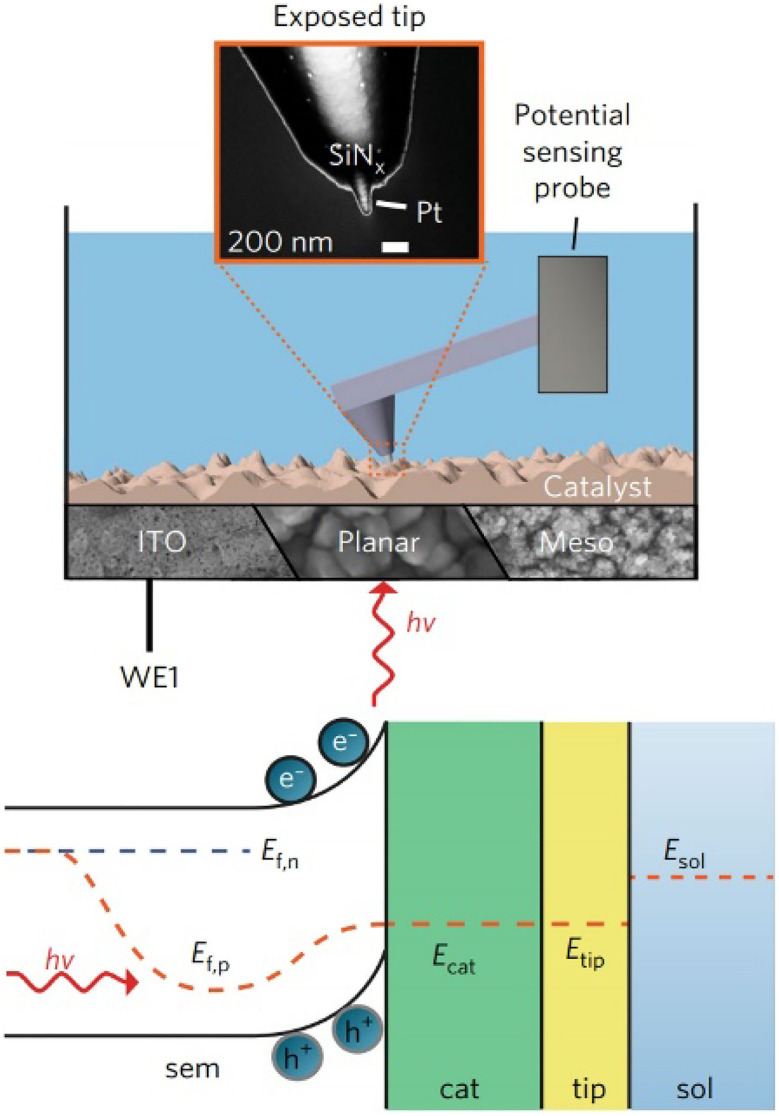
Potential-sensing electrochemical AFM setup with the cantilever, fully electrically insulated except for the exposed nanotip, serving as a potentiometric probe. The inset shows an electron micrograph of the AFM tip used. The blue color represents electrolyte. The approach allows for the study of a wide range of (photo)electrochemical structures *in operando*, including electrodeposited catalysts on conductive indium tin oxide (ITO), planar haematite and nano/mesostructured haematite, connected to the circuit *via* the working electrode (WE1). Band diagram for an illuminated semiconductor (sem) electrode. *E*_f,n_ and *E*_f,p_ are the electron and hole quasi-Fermi levels, respectively. *E*_cat_ and *E*_tip_ are the Fermi levels of the catalyst and AFM tip (tip), which are assumed to be in equilibrium. *E*_sol_ is the redox potential of the electrolyte (sol). Adapted from ref. 14 with permission from Springer Nature, copyright 2017.

*Operando* surface-potential measurement can be achieved using potential-sensing electrochemical AFM (PS-EC-AFM), which directly probes the local potential of a cocatalyst or semiconductor in electrolyte.^14,76–78^ In PS-EC-AFM, a conductive AFM-SECM probe that is electrically insulated except for an exposed nanoscale apex is brought into gentle contact with the surface, allowing the tip Fermi level to equilibrate with the local electronic structure. Under illumination, photogenerated carriers accumulated in the semiconductor/cocatalyst shift the local electrochemical potential, and the probe tracks this potential change in real time (Fig. 6). Whereas photo-SECM identifies spatial variations in reactivity through current-based feedback and photocurrent mapping, PS-EC-AFM directly reports the local CT driving force (surface/catalyst potential), enabling quantitative analysis of interfacial electronic dynamics and catalyst polarization during operation.

**Fig. 7:**
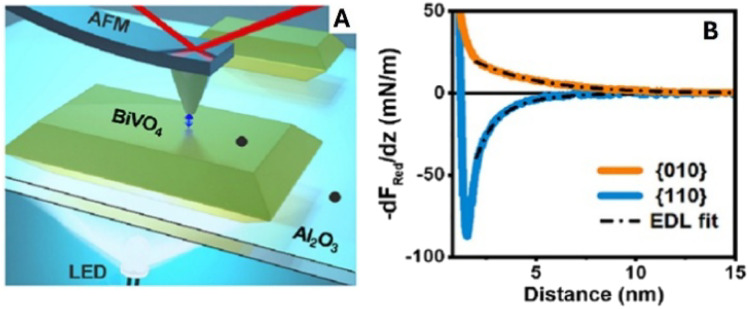
AFM force-distance measurement on BiVO_4_ particles and the photo-response in ambient electrolyte. (A) Illustration of dynamic AFM measurements in liquid with bottom illumination. (B) Average reduced interaction stiffness (*k*_red_) or force gradient (−d_Fred_/d_*z*_) *versus* distance curves across a flat region at the center of {010} and {110} facets of BiVO_4_ particles. Solid lines are experimental data after subtraction of van der Waals interaction and dashed-dotted black lines are the theoretically fitted force curves obtained using EDL theory, taking into account charge regulation. Reproduced from ref. 80 with permission from American Chemical Society, copyright 2024.

*In situ* AFM force-distance spectroscopy provides an additional, orthogonal view of light-induced interfacial phenomena by quantifying tip-sample interaction forces in electrolyte.^79,80^ The measured force gradients contain a long-range electrostatic component that depends on local surface charge and the structure of the electric double layer (EDL) (Fig. 7). Upon illumination, systematic shifts in the force–distance curves can reveal localized charge accumulation, changes in band bending, or altered screening/ion distributions at the photocatalyst–electrolyte interface. Thus, while photo-SECM visualizes redox activity *via* current response, AFM force spectroscopy interrogates the accompanying evolution of interfacial fields and electrostatic environments, providing mechanistic insight into charge migration, trapping, and accumulation under *operando* conditions.

**Fig. 8:**
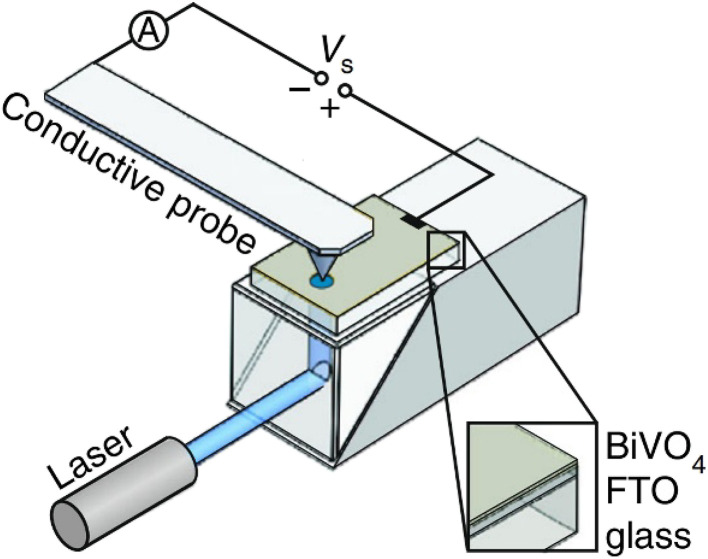
Schematic illustration of the photoconductive atomic force microscope (pc-AFM) setup. The BiVO_4_ film is illuminated from the FTO side through the transparent glass substrate. The bias voltage (*V*_s_) is applied between the metal-coated probe and the transparent FTO back electrode. Adapted from ref. 81 with permission from Springer Nature, copyright 2018.

Photoconductive AFM (pc-AFM) complements electrochemical techniques by directly mapping local photoconductivity in the solid state.^81^ As illustrated in Fig. 8, a metal-coated AFM probe contacts the semiconductor surface while a bias voltage (*V*_s_) is applied between the probe and a transparent back electrode (*e.g.*, FTO), enabling current to be recorded through the tip–sample junction. With illumination delivered through the transparent substrate, pc-AFM produces spatially resolved conductance maps and local current–voltage spectra that report on carrier generation, trapping, and transport across grains, facets, and interfaces. Because pc-AFM is performed without an electrolyte, it primarily probes solid-state carrier transport in the film and junction/contact effects at the tip–sample (and back-electrode) interfaces. In electrochemical experiments, these contributions would be entangled with interfacial CT kinetics. As a result, pc-AFM provides a useful bridge between intrinsic transport properties and *operando* photoelectrochemical activity.

**Fig. 9:**
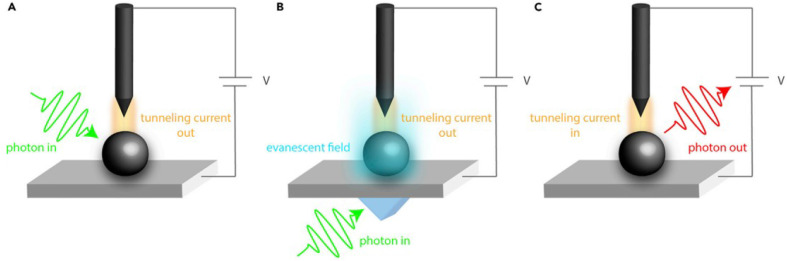
Schematic of different spectroscopic STM methods – light pump-current probe techniques (A) and (B) and a current pump-light probe technique (C). The setups used for light pump experiments: direct illumination of the tunneling junction (A) and back-illumination (B). Adapted from ref. 82 with permission from Elsevier Inc., copyright 2020.

Optical (spectroscopic) scanning tunneling microscopy (STM) techniques provide access to photoinduced electronic processes with atomic-to-nanometer spatial resolution, beyond the optical diffraction limit.^82^ In the light pump–current probe configuration (Fig. 9A and B), electromagnetic radiation is delivered into the tip–sample junction (either by direct illumination or back illumination), and the resulting changes in tunneling current reflect locally excited electronic or vibrational states. Conversely, in the current pump–light probe (STM-induced luminescence, Fig. 9C), the tunneling current or bias excites the junction and the emitted photons report on local radiative recombination pathways. Although optical STM is typically performed under ultra-high-vacuum conditions on conductive surfaces, it uniquely resolves how individual defects, dopants, and atomic-scale heterogeneities modulate absorption, hot-carrier dynamics, and recombination. These insights complement photoelectrochemical data by linking nanoscale electronic structure to macroscopic charge-transfer behavior in photo(electro)catalytic environments.

SPVM, PS-EC-AFM, *in situ* AFM force spectroscopy, pc-AFM, and optical STM can provide distinct yet complementary observables that strengthen the interpretive power of photo-SECM and other electrochemical SPMs by combining *operando* activity mapping with measurements of surface potential, interfacial electrostatics, solid-state transport, and local electronic structure at the nanoscale.

## 2 Photo-SPM studies of two-dimensional and thin-film semiconductors

Electrochemical photo-SPMs are powerful tools for studying 2D semiconductors and photocatalytic thin films, where apparent activity is often dominated by spatially localized charge separation, carrier transport, and recombination rather than by average light absorption. Spatial variations in these photophysical properties determine local surface reactivities in photocatalytic and photoelectrocatalytic systems surveyed in this section, and therefore spatially resolved photoelectrochemical measurements are essential for elucidating the effects of the support, junctions, surface steps, and defects on interfacial CT rates.

**Fig. 10:**
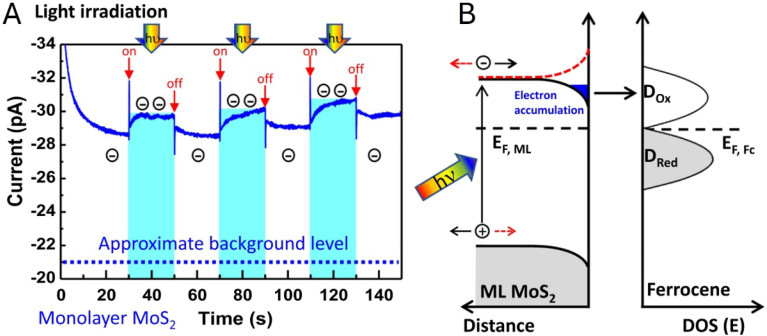
(A) Chronoamperometric *I–t* curve showing a light-driven feedback current enhancement with the probe on top of a MoS_2_ monolayer flake. At the indicated times (red arrows), a white light source (*hν* = 0.3–1.8 eV) is switched on for a period of 20 s, indicated by the bright blue areas. The current enhancement results from interface electron accumulation in response to light irradiation and downward band bending (black curve), which is not present for opposite bending (red curve) (B). Reproduced from ref. 83 with permission from Springer Nature, copyright 2021.

Chen and co-workers^83^ employed nanoscale AFM-SECM in a light-modulated chronoamperometric mode to directly probe the effect of illumination on local electron-transfer kinetics at the MoS_2_/liquid interface. With the SECM probe positioned above a MoS_2_ monolayer region, periodic white-light irradiation produced a reproducible increase in the SECM feedback current in the presence of a ferrocene mediator (Fig. 10A), indicating faster mediator regeneration under illumination. The accompanying band-diagram analysis (Fig. 10B) attributed this response to illumination-induced electron accumulation at the semiconductor/electrolyte interface under downward band bending, which raises the local electron availability for Fc^+^ reduction and thereby enhances the feedback signal.

**Fig. 11:**
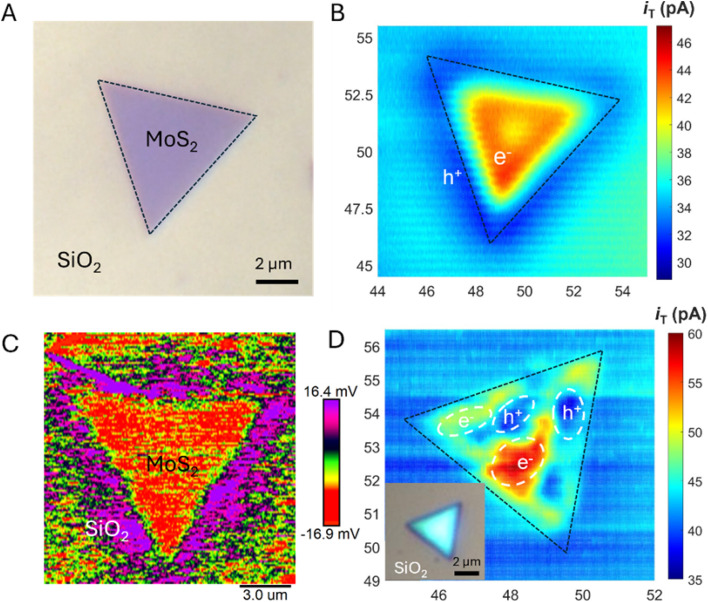
(A) Optical micrograph of a few-layer thick MoS_2_ triangle. (B) Constant-height photo-SECM image of the same triangle. (C) SPVM image of the few-layer thick MoS_2_ triangle on a SiO_2_ support in air. (D) Photo-SECM image of the bulk MoS_2_ triangle on a SiO_2_ support. Reproduced from ref. 46 with permission from American Chemical Society, copyright 2025.

High-resolution photoelectrochemical imaging of MoS_2_ triangles on a SiO_2_ support (Fig. 11A) produced the first evidence of charge separation at the semiconductor/insulator interface.^46^ The ferrocenemethanol (Fc) mediator was oxidized at the SECM tip and regenerated at the illuminated substrate, producing positive feedback (Fig. 11B) consistent with the accumulation of photogenerated electrons at the MoS_2_ surface. In contrast, when the tip was scanned over the portion of the SiO_2_ surface adjacent to the edge of the triangle, the *i*_T_ values were lower than those measured over the insulating surface (far from the triangle), pointing to redox competition between the tip and the substrate. It was suggested that the photogenerated holes migrate to the SiO_2_ surface and oxidize Fc species there. SPVM (Fig. 11C) provided an independent, non-faradaic perspective: the SPVM map revealed that photogenerated holes migrate from MoS_2_ to the SiO_2_ surface and travel laterally over distances exceeding 2 µm driven by the built-in electric field. However, in thicker and less uniform MoS_2_ nanosheets (Fig. 11D), the charge separation is dominated by internal driving forces within MoS_2_, without significant contribution from SiO_2_. These findings underscore the importance of semiconductor–support interactions for effective charge separation, suggesting a new strategy for optimizing photocatalytic systems.

**Fig. 12:**
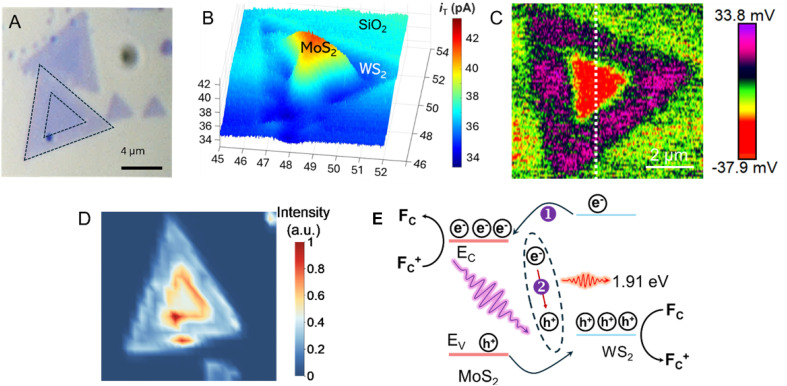
Optical micrograph (A), constant-height photo-SECM image (B), SPVM image (C), and PL image (D) of in-plane WS_2_–MoS_2_ heterojunctions. (E) Proposed energy band diagram of the heterojunction. Reproduced from ref. 84 with permission from American Chemical Society, copyright 2026.

A similar experimental approach based on the combination of photo-SECM, SPVM, and photoluminescence (PL) spectroscopy was recently used for *in situ* visualization of charge separation and recombination dynamics in monolayer MoS_2_–WS_2_ in-plane heterojunctions (Fig. 12A).^84^ The feedback-mode photo-SECM image (Fig. 12B) is conceptually similar to that in Fig. 11B, showing Fc^+^ reduction by photogenerated electrons migrating to the MoS_2_ triangle and redox competition signal pointing to Fc oxidation by photogenerated holes on the WS_2_ surface. This picture is in agreement with an SPVM image (Fig. 12C), which is consistent with directional carrier partitioning across the heterojunction. Ultraviolet photoelectron spectroscopy (UPS) was used to determine the band-edge positions of MoS_2_ and WS_2_ (valence-band maxima and work functions, with conduction-band minima inferred from the band gaps), confirming a staggered type-II alignment that drives directional interfacial charge transfer. Meanwhile, PL imaging (Fig. 12D) showed that the interface can also act as a recombination center, limiting efficient carrier extraction. The PL signal collected from the interfacial region exhibited a pronounced emission peak at ∼650 nm significantly shifted from the excitonic peaks of the individual MoS_2_ and WS_2_ monolayers, which was attributed to radiative recombination of electrons in the conduction band of MoS_2_ with holes in the valence band of WS_2_. The PL intensity map at 650 nm (Fig. 12D) reveals that the enhanced PL response is highly localized at the phase boundary, indicating that the interface acts as a localized recombination site for photogenerated electron–hole pairs, as shown in the proposed energy band diagram of the heterojunction (Fig. 12E).

**Fig. 13:**
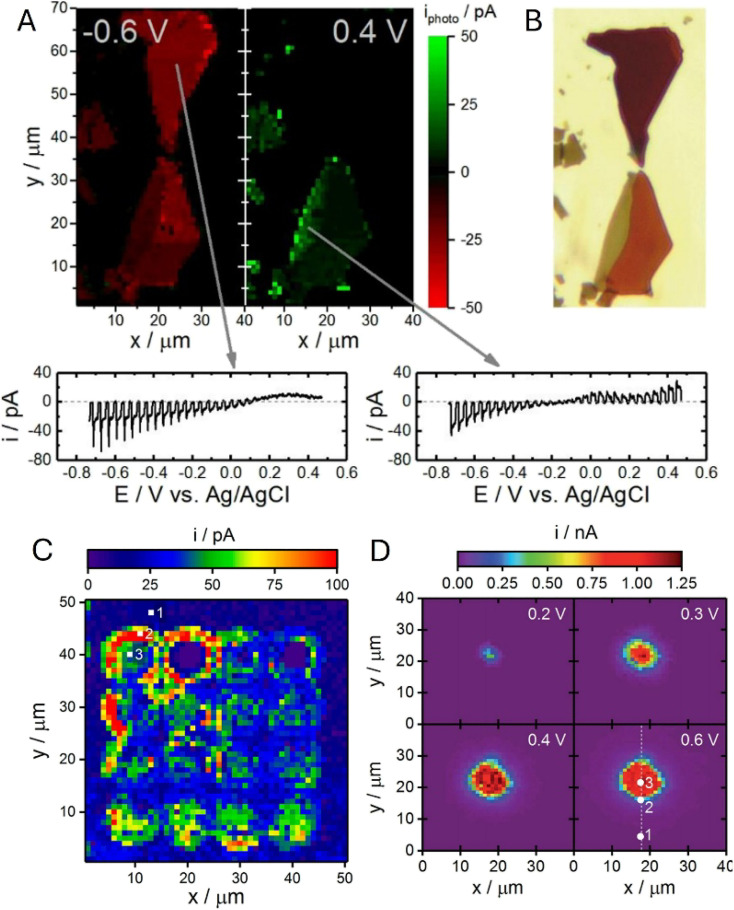
SECCM to WSe_2_ nanosheets. Correlated photoelectrochemical (A) and optical transmission (B) images of individual p-type WSe_2_ nanosheets obtained *via* SECCM. A small subset of recorded voltammograms is provided at the points indicated by grey arrows. (C) SECCM image depicting HER photocurrents at −0.96 V *vs.* Ag/AgCl across the array of anodization defects within an individual p-type WSe_2_ nanosheet. (D) CG-TC SECCM photocurrent images of carrier transport within basal planes of n-WSe_2_ nanosheets. Adapted from ref. 62, 63 and 65 with permission from American Chemical Society and Royal Society of Chemistry, copyright 2019, 2020 and 2021.

SECCM provides an alternative way to correlate local electrochemistry with a local structure by confining the reaction to a tiny meniscus contact. The Hill group applied SECCM to individual p-type WSe_2_ nanosheets, acquiring position-resolved voltammograms and converting them into spatial maps of the photoelectrochemical response (Fig. 13A).^62^ Direct comparison with the corresponding optical transmission image (Fig. 13B) showed that within a single flake, the variations in thickness and local structural features result in measurable changes in the local voltammetric response and photocurrent magnitude.

In a subsequent study, the same group used the same platform not only as a probe but also as a local modification tool.^63^ By contacting the WSe_2_ basal plane in a hopping-mode grid, they applied a double potential-step waveform through the SECCM meniscus to locally anodize the surface and generate arrays of controlled defects and subsequently performed photoelectrochemical SECCM mapping of the same patterned regions under illumination. The resulting HER photocurrent map (Fig. 13C) shows that the enhanced activity is highly localized at the engineered sites, providing a direct link between defect formation and local catalytic response.

An important advance beyond conventional SECCM mapping is carrier generation–tip collection (CG-TC) SECCM.^65^ Here, a focused laser spot generates carriers locally, while a laterally displaced SECCM meniscus collects them at a confined solid–liquid junction, so the measured current directly reflects carrier transport and loss between the generation and collection sites. CG-TC images of n-WSe_2_ nanosheets (Fig. 13D) were analyzed in conjunction with transport modeling to quantify highly anisotropic diffusion and to show that step-edge defects strongly perturb the collected signal, consistent with localized carrier recombination.

For thin-film photoelectrodes, scanning electrochemical methods are often employed to disentangle interfacial CT from electron back-transfer/surface recombination and in some cases to quantify surface intermediates. Using feedback-mode SECM, Zhang *et al.* extracted apparent hole- and electron-transfer rate constants 
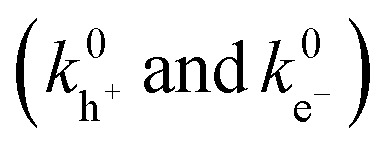
 for BiVO_4_ and Mo:BiVO_4_, showing that Mo doping substantially increases the 
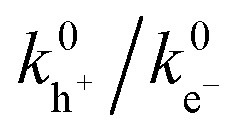
 ratio under illumination, consistent with improved interfacial charge separation.^85^ Wittstock and co-workers^86^ applied similar analysis to BiVO_4_/NiFe-LDH and concluded that the performance gain is dominated by strong suppression of electron back-transfer (reduced 
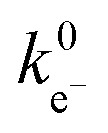
), rather than a large acceleration of surface regeneration kinetics. Additional mechanistic insights were obtained by the Bard group that used SI-SECM to quantify adsorbed OH radical intermediates on W/Mo-BiVO_4_ by photogenerating them under illumination and titrating them in the dark, thereby directly linking intermediate accumulation/decay to efficiency losses in water oxidation.^50^

**Fig. 14:**
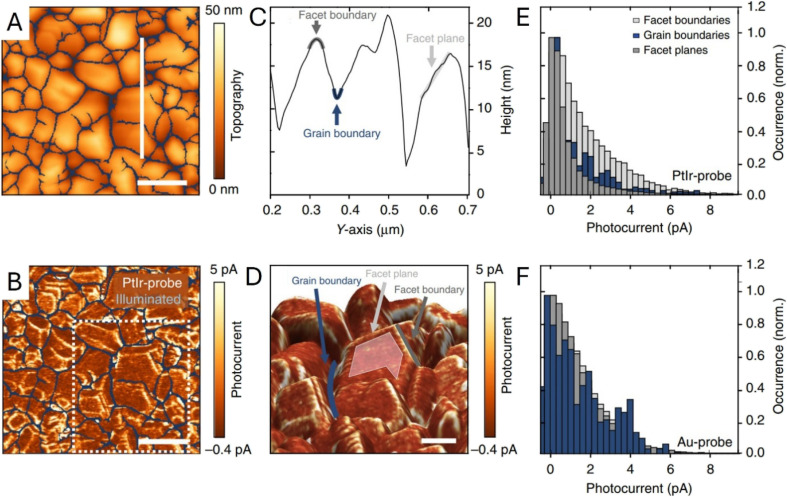
(A) Topography image and (B) photocurrent map of a BiVO_4_ thin film obtained with a PtIr-coated probe and an applied sample bias of 1.75 V. The grain boundaries are highlighted in blue. The spatially resolved photocurrent map is calculated by subtracting the current map recorded under dark conditions from that obtained under illumination. The scale bar is 200 nm. (C) Height profile along the white solid line shown in (A) defines facet planes, as well as grain and facet boundaries. (D) 3D-topography image with overlaid photocurrent contrast. A representative facet plane is highlighted in light gray, grain boundary in blue, and facet boundary in dark gray. (E and F) Histograms of the photocurrent distributions at facet planes, facet boundaries, and grain boundaries. Adapted from ref. 81 with permission from Springer Nature, copyright 2018.

Eichhorn *et al.* employed photoconductive AFM (pc-AFM) to map local, light-driven charge collection/transport in BiVO_4_ thin films with nanometer resolution, using a PtIr-coated tip to form a well-defined solid–solid contact and probe the photo-response in electrolyte.^81^ The topography image (Fig. 14A) outlines the grain structure, and the corresponding photocurrent map collected with a PtIr-coated probe under illumination (Fig. 14B) shows a highly heterogeneous photo-response across the film. A representative height profile (Fig. 14C) identifies facet planes and distinguishes facet and grain boundaries, while the 3D view with the photocurrent overlaid (Fig. 14D) makes it clear where the stronger and weaker signals occur within the microstructure. The histograms (Fig. 14E and F) quantify the photocurrent distributions and show the dependence on the probe material (PtIr *vs.* Au), emphasizing that local contact conditions can shape the apparent photocurrent landscape and should be considered when interpreting nanoscale transport maps.

**Fig. 15:**
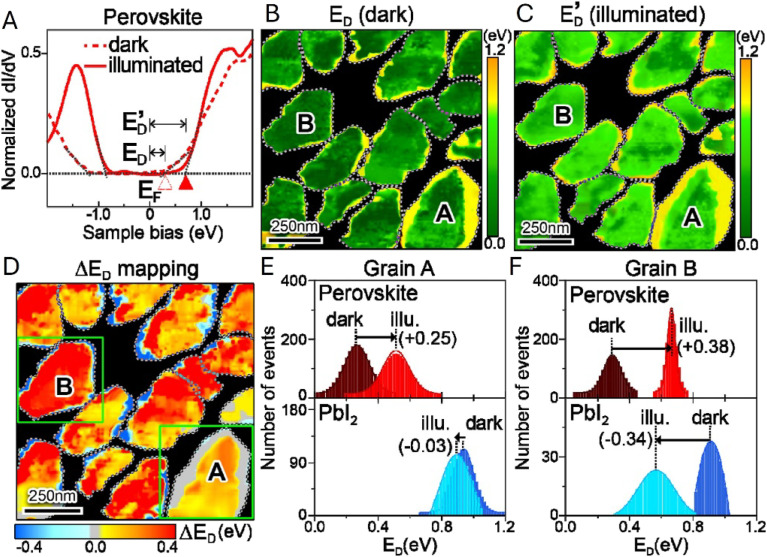
(A) Representative d*I*/d*V* curves of perovskites in the dark (dashed curve) and under illumination (solid curve) conditions. Images of the spatial distribution of the *E*_D_ and 
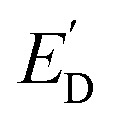
 values derived for the perovskite grains (B) in the dark and (C) under illumination. (D) Spatial distribution map of Δ*E*_D_ values 
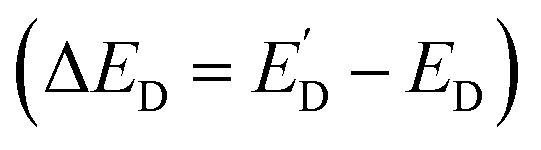
 observed for the perovskite grains under illumination. The statistical histograms of all the *E*_D_ values in grain A (E) and grain B (F) measured in the dark and under illumination, respectively. Adapted from ref. 87 with permission from American Chemical Society, copyright 2017.

Light-modulated STM/STS can be used for direct visualization of local electronic structure changes under illumination in thin-film absorbers. Shih *et al.* applied this approach to perovskite films with PbI_2_ passivation regions, using STS to track the conduction-band-edge position relative to the Fermi level (*E*_D_) in the dark and under illumination (Fig. 15).^87^ The normalized d*I*/d*V* spectra (Fig. 15A) show light-induced shifts, while the maps of *E*_D_ (dark) and 
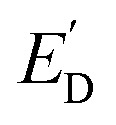
 (illuminated) (Fig. 15B and C) reveal strong intragrain heterogeneity. The 
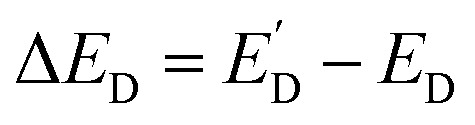
 map (Fig. 15D) highlights spatially distinct responses between grain interiors and PbI_2_-rich regions, and the histograms (Fig. 15E and F) quantify grain-to-grain differences tied to the local PbI_2_ configuration. Overall, these data show that nanoscale variations in band alignment/passivation can produce markedly different photocarrier behavior even in nominally similar grains, providing useful context for interpreting heterogeneous optoelectronic response in polycrystalline films.

The photo-SPM studies discussed above showed that local charge separation in two-dimensional and thin-film semiconductors is governed not by a single universal factor but by a combination of interfacial energetics, local structure, and carrier-transport pathways. In atomically thin materials, semiconductor–support interactions and the resulting built-in electric fields can control carrier distribution, as illustrated by the MoS_2_/SiO_2_ system, whereas in lateral heterojunctions the key driving force is the band alignment across the interface. In both SECM and SECCM measurements, structural defects, step edges, and phase boundaries frequently appear as localized recombination sites or transport bottlenecks, whereas deliberately introduced defects can enhance local activity by altering the balance between charge separation and interfacial reaction kinetics. In thicker flakes and polycrystalline thin films, the response is further shaped by thickness-dependent transport and grain and facet boundaries. These examples suggest that spatially resolved photoelectrochemical behavior emerges from the interplay of support effects, band bending, defect chemistry, and mesoscale carrier transport, underscoring the need for correlative multimodal measurements to distinguish the relative contributions of these factors.

## 3 Photo-SPM studies of photo(electro)catalysis on single particles

**Fig. 16:**
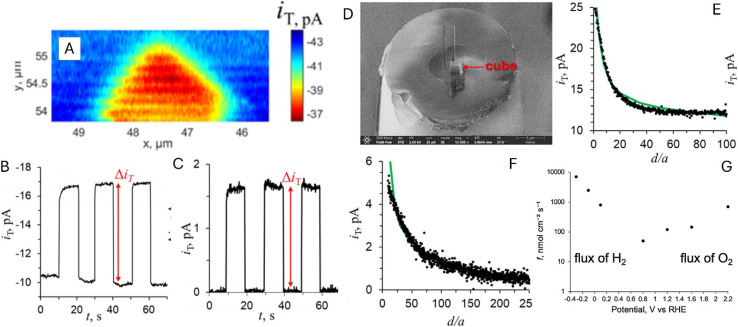
(A) 2D SECM topography image of the Al/SrTiO_3_ Rh_2−*y*_Cr_*y*_O_3_ cube attached to the glass surface. Chopped light current transients at the tip positioned over the top face of the Al/SrTiO_3_ Rh_2−*y*_Cr_*y*_O_3_ cube for the ORR (B) and the HOR (C) in a 0.1 M K_2_SO_4_ solution. (D) SEM images of the Al:SrTiO_3_ Rh_2−*y*_Cr_*y*_O_3_ cube attached to the carbon fiber microelectrode. The carbon fiber is surrounded by an insulating glass layer. (E and F) Experimental (symbols) approach curves obtained with the Pt tip over the center of the top face of the Al/SrTiO_3_ Rh_2−*y*_Cr_*y*_O_3_ crystal and fitted to the theory (solid lines). (E) *E*_S_ = 1.6 V *vs.* RHE. Calculated O_2_ flux, f_O_2__ = 145 nmol cm^−2^ s^−1^. (F) *E*_S_ = 0.1 V *vs.* RHE. Calculated H_2_ flux, f_H_2__ = 0.8 nmol cm^−2^ s^−1^. (G) Potential dependence of HER and OER rates on a single illuminated Al/SrTiO_3_ Rh_2−*y*_Cr_*y*_O_3_ cube. The solution contained 0.1 M K_2_SO_4_. Reproduced from ref. 43 and 93 with permission from American Chemical Society and Wiley, copyright 2023, 2025.

Ensemble-level studies of photo(electro)catalysts often average out strong heterogeneity across particles and facets, motivating single-entity experiments that link local photocarrier separation and transport to local photo(electro)catalysis. For example, facet-selective charge separation in BiVO_4_,^88^ visualized by SPVM^89,90^ and super-resolution imaging approaches,^91^ highlights the need to quantify local interfacial reaction fluxes *in operando*. SECM approaches pioneered by Bard and co-workers enabled *operando* screening but were resolution-limited,^35,92^ whereas recent advances in photo-SECM enabled *in situ* measurements of overall water splitting (OWS) and other photo(electro)catalytic processes on single particles. Photo-SECM was used to directly measure hydrogen and oxygen fluxes produced by OWS on individual Al:SrTiO_3_/Rh_2−*y*_Cr_*y*_O_3_ microcubes.^43^ After using feedback mode to locate a single cube (Fig. 16A), the tip was positioned over the top face to record chopped-light transients of O_2_ reduction (Fig. 16B) and H_2_ oxidation (Fig. 16C). Quantitative analysis of these transients confirmed stoichiometric H_2_/O_2_ production, providing direct evidence of OWS at a single photocatalyst particle.

To apply external bias and study photoelectrochemical processes at single particles, the same group integrated photo-SECM with microelectrode-based photoelectrochemistry by attaching an individual Al:SrTiO_3_/Rh_2−*y*_Cr_*y*_O_3_ cube to a carbon-fiber microelectrode (Fig. 16D).^93^ In this configuration, experimental approach curves were acquired for the OER (Fig. 16E) and HER (Fig. 16F) at the illuminated surface at different applied potentials and fitted to theoretical curves obtained from COMSOL simulations (green curves) to extract local O_2_ and H_2_ fluxes. The resulting potential dependence of the extracted rates (Fig. 16G) was found to agree semi-quantitatively with the dependence of the photocurrent density *vs.* the applied bias found from chopped light transients at a single cube. Within the 0–2.0 V *vs.* RHE potential range H_2_ and O_2_ rates were correlated with the cathodic and anodic photocurrents, as expected for a photocatalytic process. Outside this interval, H_2_ and O_2_ rates were much higher and mostly due to electrocatalytic generation at both the photocatalyst and the carbon fiber surface. Measurements on 20 individual photocatalyst particles showed variations in OER activity, which were attributed to different particle size and orientation on the carbon electrode. HER activity variations between catalysts were at least 10 times larger and likely caused by variable amounts of Rh_2−*y*_Cr_*y*_O_3_ catalyst on the Al:SrTiO_3_ particles. It was suggested that performance gains are possible by better controlling catalyst composition on the microscale.

**Fig. 17:**
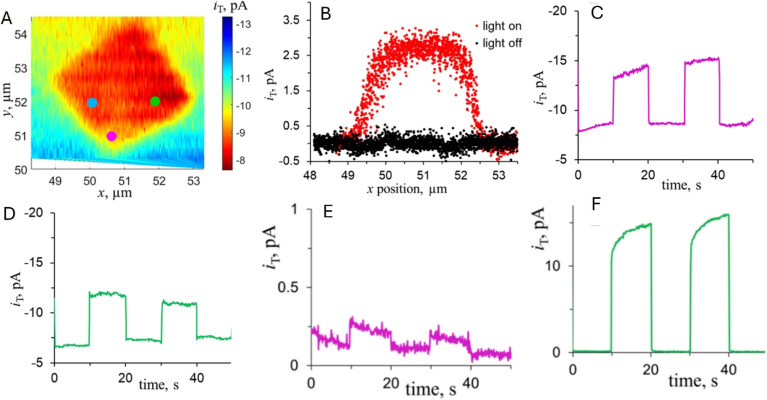
Topographic SECM image of the P:BiVO_4_ crystal (A) and lateral line scans for the HOR recorded over the P:BiVO_4_ microcrystal and underlying glass under illumination (red symbols) and in the dark (black symbols) (B). Chopped-light current transients for the ORR obtained at location 1 (C) and location 2 (D) and the HOR at locations 1 (E) and location 3 (F) shown in panel (A). Locations 1, 2, and 3 are labeled in panel A by the pink, green, and blue circles. Reproduced from ref. 44 with permission from American Chemical Society, copyright 2023.

A photo-SECM instrument equipped with a nanometer-sized tip was used to visualize the kinetic heterogeneity of OWS on individual truncated bipyramidal P:BiVO_4_ microcrystals.^44^ After locating a crystal by feedback-mode SECM topography imaging (Fig. 17A), the tip was scanned over its top facet to detect the HER under illumination (red symbols in Fig. 17B). Local chopped-light transients for O_2_ and H_2_ detection (Fig. 17C and D) were recorded at the tip positioned over three interrogation sites shown in Fig. 17A. These transients showed major variations in HER and OER rates over different areas of the same crystal consistent with previously reported separation of photogenerated electrons and holes on different crystal facets of BiVO_4_.^88–91^ However, a smaller tip size and higher spatial resolution are required to exactly localize the facet boundaries and map the photocatalytic activity within the near-edge transition zones.

The possibility of multi-technique (*i.e.*, photo-SECM/TEM and photo-SECM/AFM) imaging aimed at correlating photo-SECM maps with high-resolution structural data was explored by using TEM finder grids as a conductive support for individual TiO_2_ nanorods.^41^

**Fig. 18:**
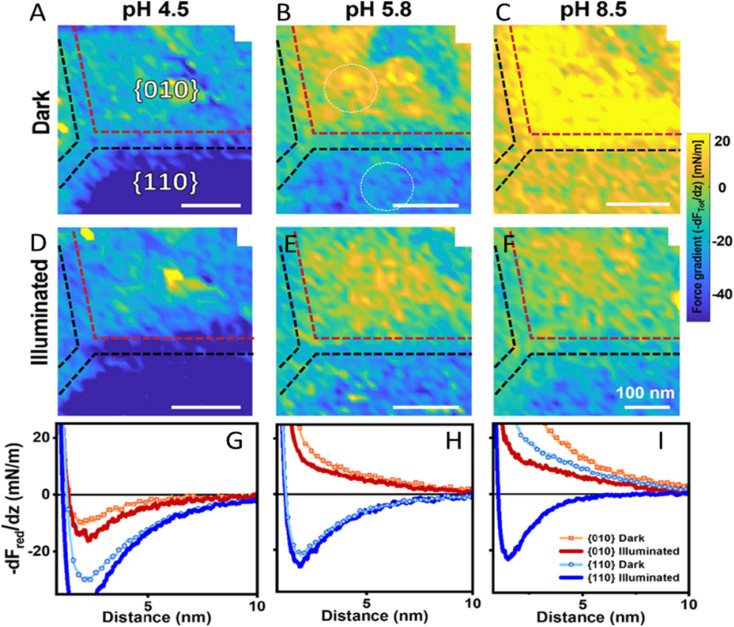
Facet-resolved photo-response of an individual BiVO_4_ NP at variable pH. (A)–(F): force gradient (−d*F*_Tot_/d*z*) maps at 2.5 nm above the BiVO_4_ surface under dark and illumination conditions and pH values as indicated (NaCl concentration: 10 mM). (G)–(I): reduced force gradient (−d*F*_red_/d*z*) curves with and without illumination for both facets. Panels (A)–(F) show the total force gradient as measured, whereas panels (G)–(I) show the reduced force gradient after subtraction of the van der Waals attraction. Adapted from ref. 80 with permission from American Chemical Society, copyright 2024.

Closely related to kinetic heterogeneity mapping is the role of solid–liquid interfacial electric fields. The electrolyte can strongly influence the selectivity of nanoparticle (NP) facets because surface charging and double-layer fields are highly pH dependent. Using *in situ* AFM force-gradient mapping on a single BiVO_4_ NP, Su *et al.* showed that the contrast between {010} and {110} facets evolves with pH in the dark (Fig. 18A–C, pH 4.5/5.8/8.5) and changes further upon illumination (Fig. 18D–F), indicating a facet- and pH-dependent photo-response of the local surface potential in electrolyte.^80^ The corresponding reduced force-gradient curves (Fig. 18G–I) quantify these trends by directly comparing dark and illuminated responses for both facets at each pH. Localized defect/step regions within the maps exhibit photo-responses distinct from adjacent terrace areas, consistent with electrostatic “hotspots” that can locally modify interfacial fields and influence charge separation.

**Fig. 19:**
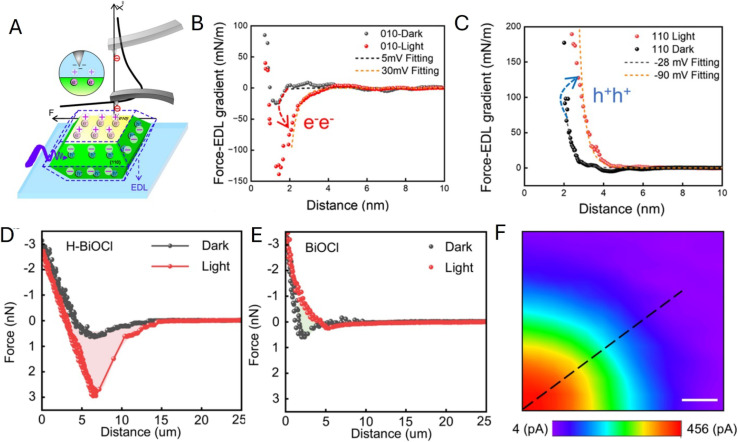
(A) Experimental setup for force curves and schematic illustration of surface charge distribution of an individual BiVO_4_ NP in the electrolyte. (B and C) Force gradient curves of the {010} facet and {110} facet in the dark state (dark) and under illumination (red). Arrows indicate the transition from the dark to the light state. (D and E) Force curve of an individual H-BiOCl NP (D) and BiOCl NP (E) at the center of the top facet in the dark and under illumination for surface charge measurement. (F) Scanning photoelectrochemical microscopy image of part of an H-BiOCl NP. Reproduced from ref. 94 and 95 with permission from American Chemical Society, copyright 2025, 2026.

In a related study, Fan and co-workers showed that changing the reaction environment, including pH, can flip the direction of the effective solid–liquid interfacial electric field on BiVO_4_ facets, thereby changing the facet capacities for delivering electrons and holes and redistributing charge-transfer activity on a single NP.^94^ The force-curve analysis (Fig. 19A) was used to extract local surface potential values from electrostatic interactions measured in electrolyte. The force *vs.* EDL gradient curves obtained in the dark (black symbols) and under illumination (red symbols) for the {010} facet in Fig. 19B and {110} in Fig. 19C were fitted to theory (dashed curves) to evaluate facet-specific, light-induced changes in surface potential/charging and provide a quantitative picture of environment-controlled field reversal.

The same group demonstrated interface tuning by surface chemistry by comparing hydroxylated H-BiOCl and pristine BiOCl NPs.^95^Fig. 19D–F show markedly different light/dark force curves obtained at these particles and a scanning photoelectrochemical microscopy map of Ru(NH_3_)^3+^ reduction on an H-BiOCl NP under illumination.

**Fig. 20:**
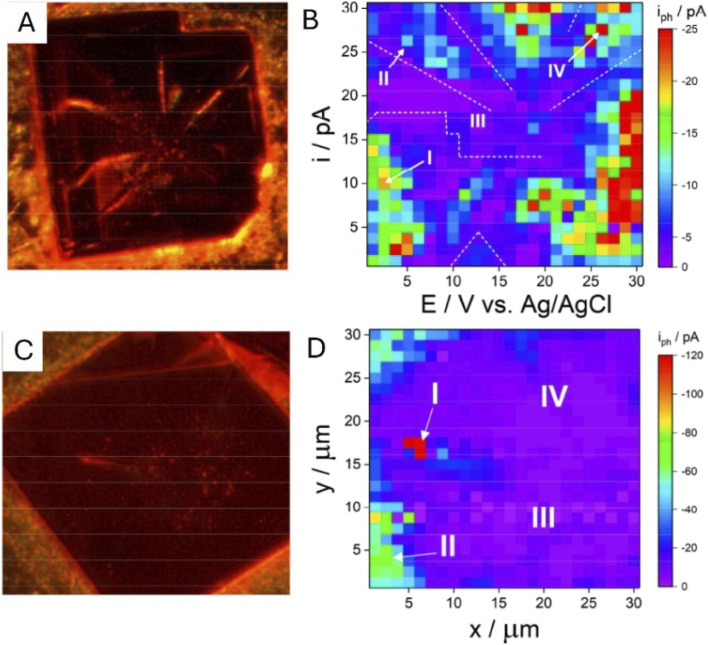
Correlated optical and SECCM photocurrent images of highly faceted (A and B) and smooth (C and D) MAPbBr_3_ crystals. Reproduced from ref. 96 with permission from American Chemical Society, copyright 2023.

Saha *et al.* mapped photocurrent responses on methylammonium lead bromide (MAPbBr_3_) single crystals with sub-micron resolution by single-entity SECCM imaging with a ∼200–500 nm nanopipette probe.^96^ In highly faceted systems, the correlated optical and SECCM maps (Fig. 20A and B) show strong heterogeneity, and the regions associated with facet boundaries and other structural defects exhibit low photocurrents consistent with enhanced carrier recombination at these features. In contrast, the flatter crystals (Fig. 20C and D) display a more homogeneous photocurrent distribution with somewhat higher currents near crystal edges, likely reflecting local variations in crystal-substrate contact.

## 4 Probing photocatalysts using the tunneling mode of photo-SECM and potential-sensing SPMs

**Fig. 21:**
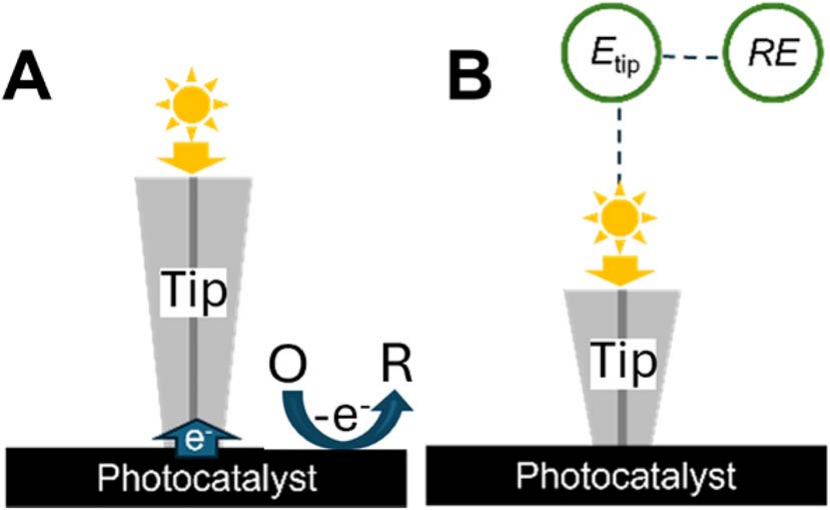
Schematic representation of the amperometric tunneling and potentiometric modes of photo-SECM operation. (A) Tunneling mode: the source of the tip current is the charge transfer occurring at the photocatalyst/solution interface. (B) Local surface potential is measured in the contact potentiometric mode.

Conventional (*i.e.*, diffusion-based) photo-SECM cannot directly probe surface potentials and driving forces for charge-transfer processes, and its spatial resolution is fundamentally limited by diffusion within the tip–substrate gap. The methods surveyed in this section address both these limitations. In the tunneling mode of photo-SECM,^97^ a nanoelectrode is brought within ∼1 nm from the sample surface to form a tunneling junction (Fig. 21). At such short separation distances, a conductive specimen (*e.g.*, a metal NP^98^) acts as a part of the nanotip, so that voltammograms of such a sample can be obtained without attaching it to the tip surface. The tip applies an electric bias to the sample surface, and the charge-transfer process occurring at the photocatalyst/solution interface is the source of the tunneling current that flows between the tip and the photocatalyst (Fig. 21A). This approach enabled electrochemical interrogation of individual NPs and electrocatalytic sites and topography mapping with higher resolution than that attainable with conventional diffusion-based SECM.^97,99^

**Fig. 22:**
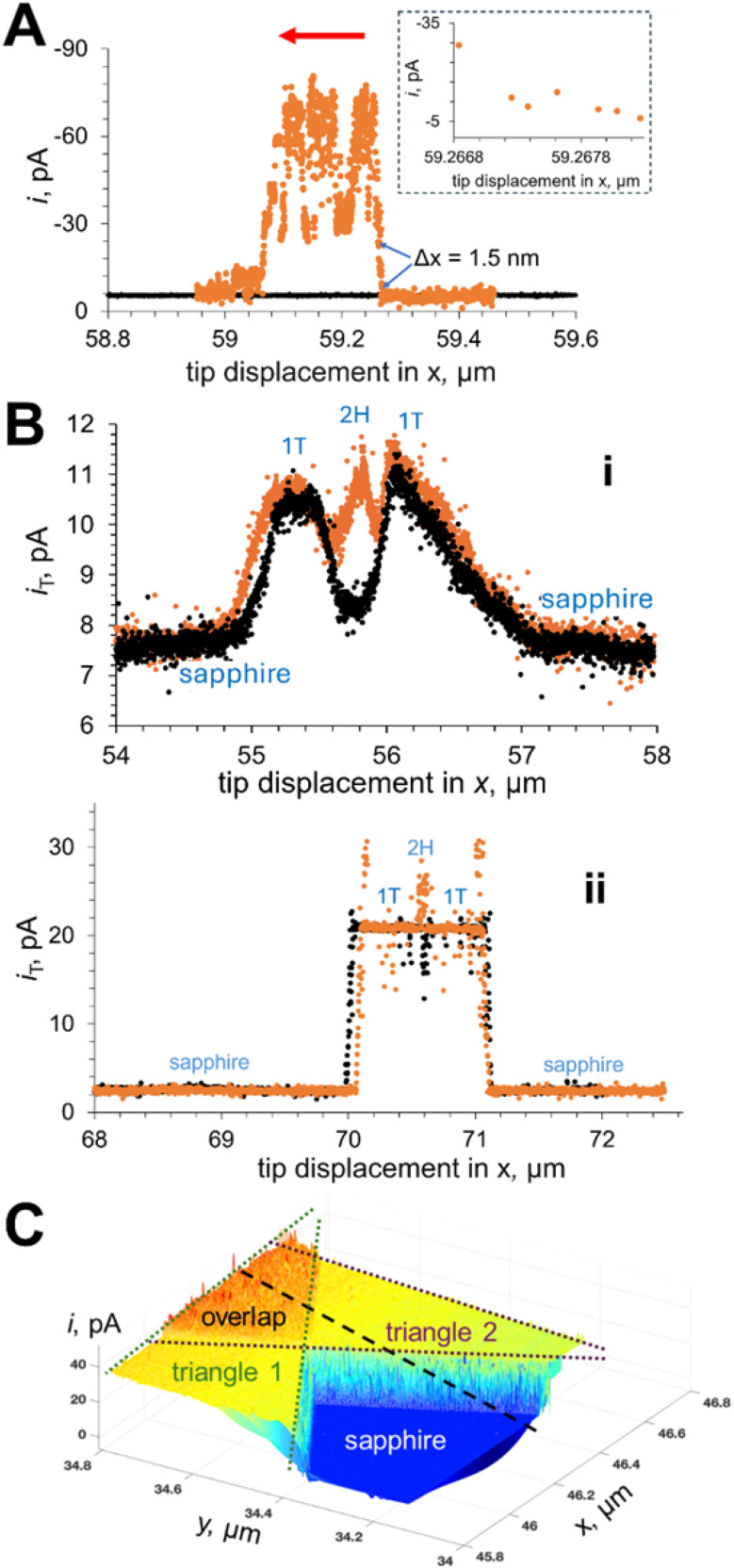
Imaging MoS_2_ triangles (A and C) and mixed phase nanosheets (B) using the tunneling mode of photo-SECM. (A) Tunneling mode lateral scans over a MoS_2_ monolayer triangle on the sapphire support recorded in the absence of light (black curve) and under illumination (orange). (B) Feedback (i) and tunneling mode (ii) lateral scans over mixed-phase MoS_2_ nanosheets in the dark (black) and under illumination (orange). (C) Colored 3D tunneling photo-SECM image of partially overlapping monolayer triangles with green and purple dotted lines showing the areas of two triangles and the orange region corresponding to their overlap. The 0.1 M KCl solution contained (A) 1 mM HClO_4_, (B) 1 mM K_4_Fe(CN)_6_, and (C) 1 mM ferrocenemethanol (Fc). Tip radius, *a* = 45 nm. *E*_T_, V *vs.* Ag/AgCl = −0.7 (A), 0.4 (B and C). Adapted from ref. 97 with permission from American Chemical Society, copyright 2025.

The lateral resolution demonstrated in tunneling photo-SECM experiments was as high as ∼1 nm. Two line scans over the edge of a MoS_2_ monolayer triangle (Fig. 22A) were recorded in the dark (black curve) and under UV-Vis illumination (orange curve). In the absence of light, the MoS_2_ monolayer triangle is electrochemically inert, the line scan is featureless, and the tip current is much lower than its bulk value (*i*_T,∞_ = 19 pA). By contrast, the current drastically increased when the Pt tip was moved from the sapphire support to the irradiated 2H MoS_2_ surface (orange curve). The tip displacement corresponding to this change was *ca.* 1–2 nm, and the *i*_T_ increased essentially monotonically within the 1.5 nm displacement (the inset in Fig. 22A).

A major enhancement of spatial resolution can be seen by comparing the lateral scans over mixed phase MoS_2_ nanosheets with the photocurrent due to the positive SECM feedback (Fig. 22B(i)) and tunneling (Fig. 22B(ii)). The resolution of feedback mode photo-SECM imaging is determined by the tip radius (∼40 nm in Fig. 22B(i)). The higher spatial resolution of tunneling photo-SECM (Fig. 22B(ii)) resulted in much sharper current changes corresponding to transitions between different surfaces and allowed for visualization of a narrow (∼50 nm wide) region of 2H MoS_2_ (redox active under illumination and inert in the dark) located between two T1 regions (equally active in the dark and under illumination). It would not be impossible to detect such a region by feedback mode SECM with a similarly sized tip.

In a tunneling photo-SECM image (Fig. 22C), one can clearly distinguish between a single layer triangle and a bilayer formed by two overlapping MoS_2_ triangles based on the differences in the lateral charge-transfer rate. These results establish tunneling photo-SECM as a powerful tool for resolving catalytic and electronic heterogeneities in 2D semiconductors at the single nm scale. However, the factors determining the magnitude of the tunneling current have not yet been elucidated. Although the source of tunneling current in Fig. 22C is the diffusion of ferrocenemethanol to the MoS_2_ surface and its photooxidation there, the recorded *i*_T_ is about two orders of magnitude lower than the expected nA-range steady-state diffusion-limited current of 1 mM Fc to an electrode shaped as a unilateral triangle with an ∼10 µm side length. In contrast, the tunneling current measured with metal nanoparticles was close to the diffusion-limited current of the redox mediator to a NP surface.^98^ It was suggested that the lateral carrier transport to the tip in the MoS_2_ layer may limit the tunneling current.

**Fig. 23:**
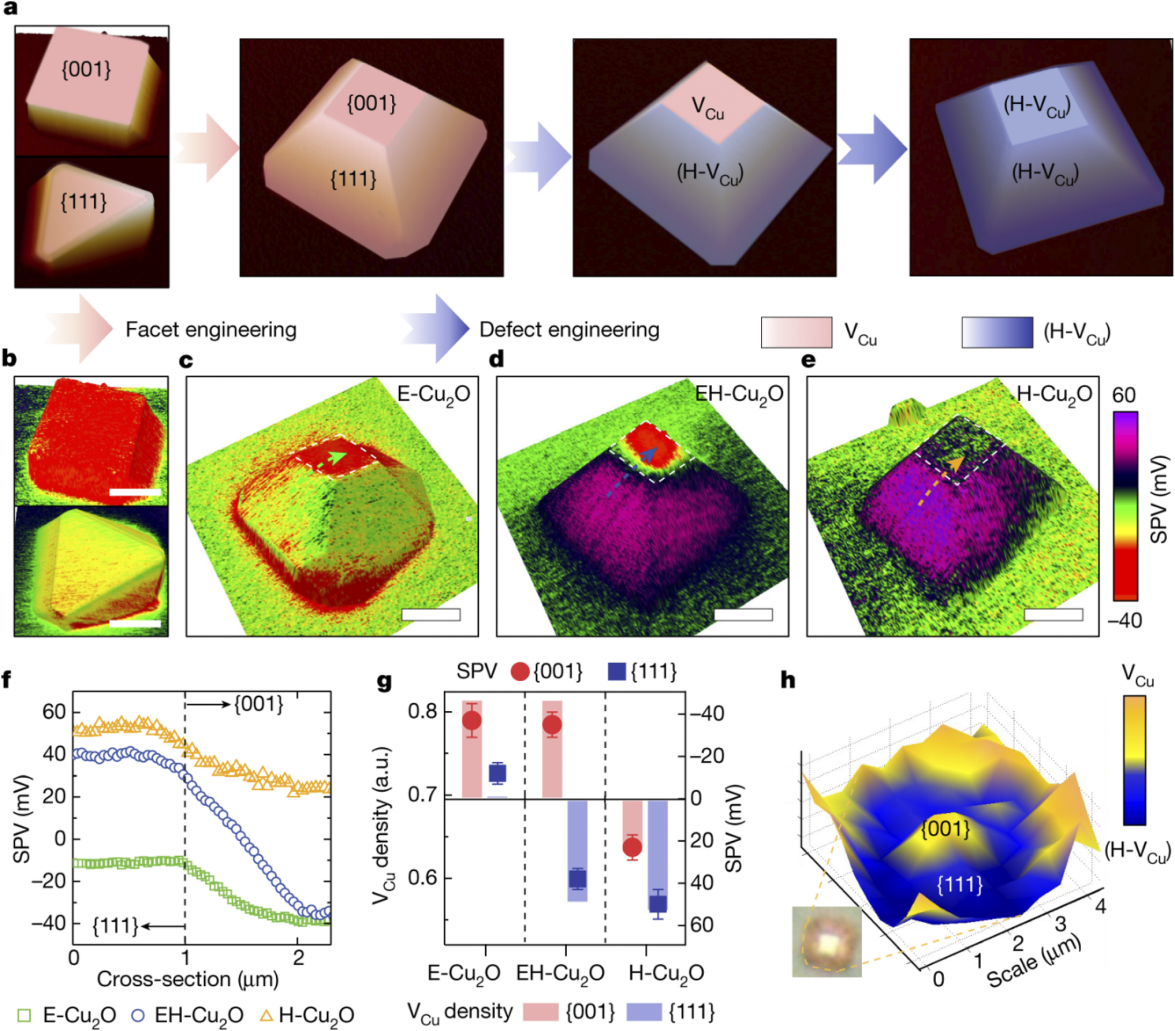
(a) Illustration of the anisotropic engineering of facets and defects of Cu_2_O photocatalyst particles. (b) SPVM images of cubic (top) and octahedral (bottom) Cu_2_O particles. (c–e) SPVM images of truncated octahedral Cu_2_O particles without (c), with moderate (d) and with extreme (e) incorporation of (H-V_Cu_) defects. Scale bars in b–e: 2 µm. Dashed arrows in panels c–e denote the positions for extracting SPV distributions across {111} and {001} facets. (f) SPV values extracted across dashed lines in panels c–e. (g) Correlations between the V_Cu_ density and SPV. (h) Confocal Raman microscopy image of an EH-Cu_2_O particle mapped with the V_Cu_-related Raman peak intensity. The low Raman intensity denotes H-V_Cu_, which compensates for V_Cu_. Reproduced from ref. 75 with permission from Springer Nature, copyright 2022.

Different SPMs typically based on Kelvin probe force microscopy (KPFM)^75^ have been used to measure the local surface potential of photocatalysts in air. Several publications by Li, Fan and coworkers are focused on the development of different modes of surface photovoltage microscopy (SPVM) and their applications to spatiotemporally resolved surface photovoltage measurements in single photocatalytic particles and nanostructured photocatalysts.^12,47,75^ An impressive example is SPVM mapping of charge transfer processes in cuprous oxide photocatalyst particles at the single-particle level.^75^ The authors used SPVM to relate anisotropic structures in Cu_2_O photocatalyst particles to surface charge distribution (Fig. 23). Facet engineering was used to tune the facet ratio of Cu_2_O particles and change their morphology from cubic to octahedral (Fig. 23a). From SPVM maps of the surface charge distribution on these particles, it was found that more photogenerated electrons are accumulated on the {001} facet than on the {111} facet of the octahedron (Fig. 23b).

Spatially controllable defect engineering (Fig. 23a) was performed to selectively extract holes onto the {111} surface of copper oxide particles and maintain electron transfer to their {001} surfaces. After defect engineering, SPVM showed that the moderate incorporation of hydrogen-compensated copper vacancy defects (H-VCu) resulted in efficient spatial separation of photogenerated electrons and holes on the {111} and {001} facets, respectively (Fig. 23d), whereas the extreme (H-VCu) incorporation led to the disappearance of surface electrons and the spreading of holes over the entire particle surface (Fig. 23e). Quantitative comparison of the SPV distributions across the {001} and {111} facets of the three particles (Fig. 23f) suggested effective charge separation on the EH-Cu_2_O surface through anisotropic defect engineering. These results, combined with confocal Raman microscopy probing of non-uniform defect distributions (Fig. 23h), indicate that anisotropic defects contribute to efficient charge separation on the photocatalyst surface.

SPMs, including SECM^100^ and gap-distance modulation method,^101^ have previously been used for surface potential measurements in solution. The Boettcher group demonstrated the possibility of making nanoscale *operando* measurements of surface potentials under practical photoelectrochemical conditions by potential-sensing electrochemical AFM (PS-EC-AFM),^14,102^ enabling potential mapping at catalyst surfaces. Their experiments showed the ability of PS-EC-AFM to spatially resolve the electronic properties of semiconductor/catalyst nanointerfaces. Under illumination, the changes in local surface potential reveal where electrons and holes accumulate, how strongly they are separated, and what overpotential is available for a given half-reaction.^14^ Because charge-transfer rates depend strongly on the local overpotential, even modest spatial variations in surface potential can result in large differences in local photocatalytic activity. Measuring surface potentials in solution is, therefore, essential for quantifying the true interfacial driving force on working photocatalysts and for linking nanoscale energetics to catalyst performance.^103^

**Fig. 24:**
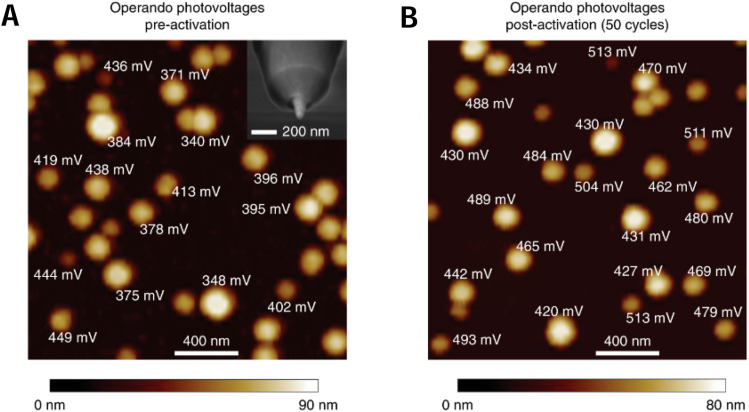
PS-EC-AFM of n-Si/Ni photoelectrodes. (A) *Operando* photovoltages collected using PS-EC-AFM before extended electrochemical activation, by landing the AFM tip on individual nanocontacts. The inset shows an SEM image of the PS-EC-AFM Pt nanoelectrode tip. (B) *Operando* photovoltages recorded at a different location after cycling the electrode 50 times under illumination from −0.35 to 0.35 V *versus E*_O_2_/OH^−^_. Adapted from ref. 102 with permission from Springer Nature, copyright 2020.

Using nominally hemispherical Ni nanocontacts electrodeposited onto n-Si as a model system, Laskowski *et al.*^102^ probed interfacial electron-transfer processes and mapped the photovoltage generated during photoelectrochemical oxygen evolution at nanoscopic semiconductor/catalyst interfaces. After topography imaging of the surface, the photovoltage, *i.e.*, the difference between the measured AFM tip potential and the back potential applied to the semiconductor ohmic contact, was measured on individual n-Si/Ni/NiOOH junctions by PS-EC-AFM. Photovoltages were collected both before (Fig. 24A) and after (Fig. 24B) 50 photoanodic voltammetric cycles. *Operando* photovoltages were substantially higher (in some cases, >500 mV) than those measured under dry *ex situ* conditions. The photovoltages dramatically increased after photoelectrochemical potential cycling and showed a strong dependence on nanocontact size. These results pointed to a size-dependent mechanism for increasing the Ni-nanocontact carrier selectivity that is operative only in the presence of the OER-active NiOOH surface layer.

**Fig. 25:**
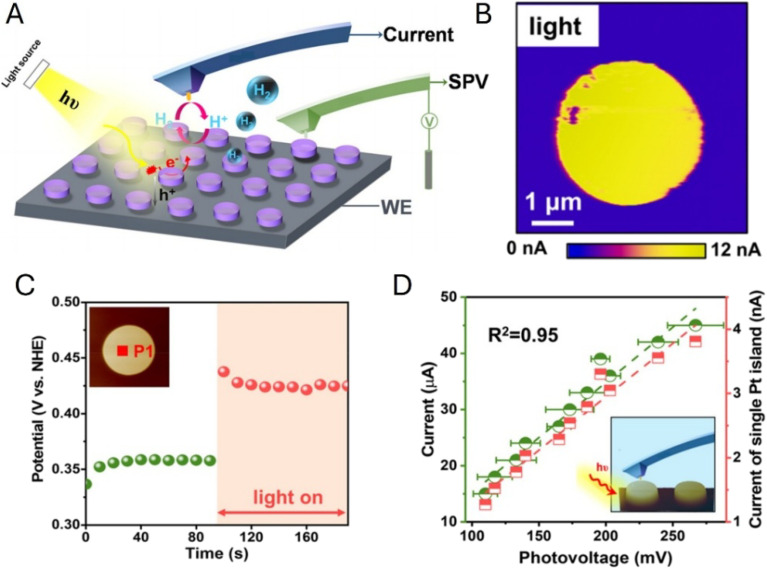
(A) Schematic of the *operando* scanning photocurrent and photovoltage measurements at the Pt/Ti electrocatalyst array on the p-Si based photoelectrode. The photocurrent images were obtained using the sample generation–tip collection in the lift mode, and the photovoltage signals were measured in the contact mode. (B) HER image of local hydrogen evolution at −0.3 V. (C) Surface electrochemical potential variation measured on a single Pt/Ti island after light excitation. (D) Relationship between SPV values measured on a single Pt/Ti island and the HER current of the photoelectrode and a single Pt/Ti island. Reproduced from ref. 104 with permission from American Chemical Society, copyright 2021.

Fan and coworker employed an AFM-based probe to investigate Pt/Ti island arrays on p-Si (Fig. 25A).^104^ They measured the surface electrochemical potential in solution using a constant-current (chronopotentiometric) protocol under dark and illuminated conditions and obtained the SPV value as the difference between the two potentials (Fig. 25C). This approach is different from PS-EC-AFM,^102^ which monitors the illumination-induced surface-potential response under more realistic conditions, *i.e.*, without passing a constant current through the sample. Since SPV reflects illumination-induced interfacial charge accumulation (*i.e.*, the local photovoltage response), it was correlated with the HER activity mapped in the sample generation–tip collection mode (Fig. 25B). A strong linear correlation was observed between the local HER current density and the measured photovoltage at individual catalytic sites (Fig. 25D), establishing a quantitative link between interfacial charge accumulation and reaction current at the nanoscale.

Unlike other methods surveyed above, the recently introduced amperometric/potentiometric mode of photo-SECM employs a single tip capable of performing both amperometric and potentiometric measurements to simultaneously probe local product fluxes and surface potentials of photocatalysts.^105^ When an SECM probe is brought within the tunneling distance from the sample surface, a conductive specimen acts as a part of the nanotip (Fig. 21A), and the local surface potential can be measured by switching from the amperometric to the potentiometric mode (Fig. 21B). Importantly, feedback mode, SG/TC, and potentiometric SECM experiments can be carried out with the same nanotip and over the same nanoscopic sample area to obtain spatially resolved information about charge separation, photovoltage, and photocatalytic activity.

**Fig. 26:**
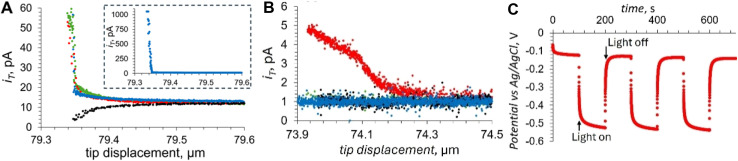
Probing the Pt/TiO_2_ photocatalyst with a photo-SECM operated in an amperometric/potentiometric mode. Approach curves (A and B) were recorded with the same tip over the Pt particle in the dark (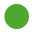) and under illumination (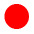) and over the TiO_2_ surface in the dark (•) and under illumination (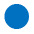). The inset in panel A shows the transition from feedback current to tunneling at a short tip/TiO_2_ distance under illumination. (C) Chopped light transients obtained with the same tip positioned within the tunneling distance from the same Pt NP. *a* = 30 nm. *E*_T_ = 0.4 V *vs.* Ag/AgCl. The solution was 0.1 M KCl containing 1 mM K_4_Fe(CN)_6_ and 10 µM K_3_Fe(CN)_6_ (A) or 0.1 M PB, pH 7 (B and C). Adapted from ref. 105 with permission from National Academy of Sciences, copyright 2026.

Nb-doped TiO_2_ rutile (110) single crystals with electrodeposited Pt NPs were used as a model system to develop and validate potentiometric/amperometric photo-SECM methodology.^105^ In the feedback mode, a Pt nanotip serving simultaneously as a nanoelectrode and a light guide for through-tip illumination approached either a Pt NP or the TiO_2_ surface (Fig. 26A). The approach curves recorded above the particle center in the dark (green) and under illumination (red) both showed strong positive feedback, confirming efficient regeneration of ferrocyanide at the Pt surface. In contrast, curves recorded over a nearby TiO_2_ region exhibited negative feedback in the dark (black), which switched to positive feedback under illumination (blue), indicating that ferricyanide reduction occurs at illuminated TiO_2_ but not in the dark. The sharp current increase at short tip–sample distances (inset in Fig. 26A) is due to the onset of tunneling.

When the electrolyte that contained a redox mediator was replaced with the phosphate buffer (PB) solution, the approach curve recorded at the positively biased tip above a Pt NP under illumination (red symbols in Fig. 26B) showed a significant HOR current at the tip, whereas the curves acquired over adjacent TiO_2_ showed no measurable hydrogen flux. Thus, the photocatalytic HER occurred exclusively on the Pt cocatalyst. The corresponding chopped-light potentiometric transients obtained with the same tip held within the tunneling distance from the same Pt NP (Fig. 26C) revealed a pronounced cathodic shift of the *E*_T_ upon illumination consistent with electron accumulation on Pt and providing the local driving force for HER.

**Fig. 27:**
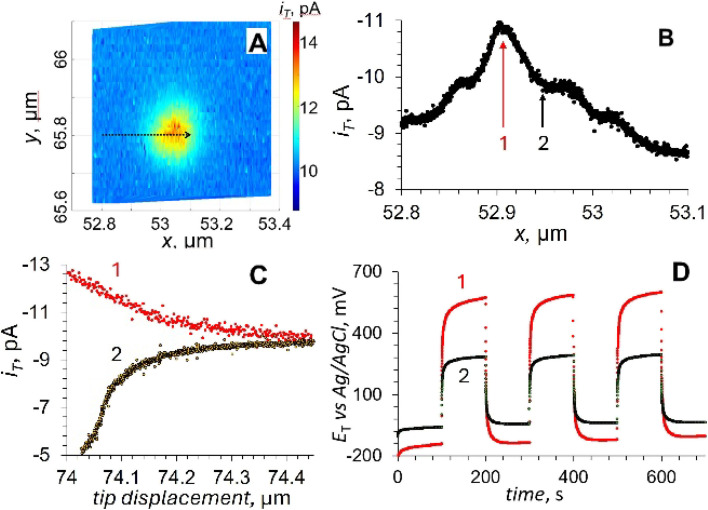
(A) Topography image of a Pt NP on the TiO_2_ surface. (B) Lateral scan recorded under illumination over the trajectory shown by the arrow in panel A. (C) Approach curves obtained under illumination at locations 1 and 2 shown in panel B. (D) Chopped light potential transients obtained with the same tip positioned within the tunneling distance from the TiO_2_ surface at the same locations 1 and 2. The solution contained 1 mM K_4_Fe(CN)_6_ and 10 µm K_3_Fe(CN)_6_ in 0.1 M KCl (A) or 0.1 M PB (B–D). *a* = 30 nm. *E*_T_, V *vs.* Ag/AgCl = 0.4 (A) and f0.7 (B–D). Adapted from ref. 105 with permission from National Academy of Sciences, copyright 2026.

The spatial localization of the OER was more complicated. A feedback mode topography image of a Pt NP on TiO_2_ was used to select a trajectory for a line scan (an arrow in Fig. 27A). The tip was scanned laterally across the Pt/TiO_2_ boundary under illumination (Fig. 27B), and the recorded ORR current showed a peak (location 1), corresponding to a catalytic hotspot for oxygen evolution on the TiO_2_ surface. This hotspot is located ∼100 nm away from the Pt NP edge, while a very low ORR current (at the background level due to O_2_ dissolved in solution) was recorded closer to the Pt NP (location 2) and over the NP surface.

The approach curves acquired at locations 1 and 2 (Fig. 27C) also suggest that the OER only occurs on the TiO_2_ surface at a certain distance from the Pt NP. This finding was corroborated by potentiometric transients recorded at the same locations with the tip positioned within the tunneling distance from the sample surface (Fig. 27D). A large positive photovoltage provides the driving force for the ORR at location 1 in contrast to a much smaller potential change at location 2. Amperometric/potentiometric SECM thus directly reveals the spatially resolved charge separation and the associated *operando* potential landscape for OWS. Scanning electron microscopy images coupled with energy-dispersive X-ray spectroscopy (SEM-EDX) and XPS Pt 4f spectra of Pt/Nb:TiO_2_ photocatalysts were obtained to interpret these results. It was found that in addition to larger metallic Pt NPs, which retain a high work function during OWS and act as electron-rich cathodic sites, ultrathin Pt patches (formed during the initial electrodeposition of the Pt catalyst on the TiO_2_ surface) get oxidized and serve as anodic sites for oxygen evolution due to their lower work function. Holes photogenerated in Nb:TiO_2_ possess limited mobility and therefore preferentially accumulate within the electron-depleted zones surrounding Pt NP, producing OER hotspots. Future advances can be made by correlating potential and flux maps, interpreting them through transport/kinetic modeling, and extending this approach to practically important semiconductor/cocatalyst systems.

## 5 Summary and future outlook

The rapid recent progress in nanoscale photo-SECM and related techniques opened new avenues for studies of nanostructured photocatalysts. Using a multimodal SECM approach that combines surface potentiometry with SG/TC-based flux mapping, one can directly map the steady-state energetic landscape along with the spatial distribution of reaction rates on individual catalytic domains under *operando* conditions. Measuring local activity and local driving force on the same microscopic sample area enables distinguishing the contributions of various factors to photocatalyst performance. Nevertheless, no single technique can fully resolve the coupled processes of light absorption, charge separation, carrier transport, interfacial charge transfer, and photocatalytic activity. Photo-SECM and complementary SPM approaches such as surface photovoltage microscopy, potential-sensing methods, AFM-based force spectroscopy, photoconductive AFM, and optical STM provide access to local energetics, interfacial fields, carrier dynamics, and electronic structure. Their future development, especially in multimodal and *operando* workflows, should enable more direct correlations between local activity, driving force, and material properties. An additional opportunity lies in the development of hybrid and next-generation probe platforms. Emerging efforts to integrate electrochemical probes with optical and spectroscopic functionality may help overcome current trade-offs between resolution, versatility, and quantitative rigor.

The transition from qualitative activity mapping to reliable quantitative analysis is essential for making electrochemical photo-SPMs more useful for studies of photo(electro)catalysis. In photo-SECM and photo-SECCM experiments, data analysis is often challenging because of many factors contributing to the measured signal, such as local interfacial kinetics, optical heterogeneity, non-local generation of reactive species, mass transport, and uncertainties in probe geometry or meniscus contact area. Future progress will therefore require better-defined illumination conditions, improved calibration protocols, and tighter integration with transport and kinetic modeling to enable extraction of meaningful quantitative information in addition to relative activity contrast. A promising approach is to employ AI for autonomous measurements, signal extraction, and building digital twin models of operating photocatalysts.^106^

Another challenge is to combine high spatial resolution with meaningful temporal resolution. Key photoelectrochemical processes, including charge separation, trapping, recombination, intermediate accumulation, and catalyst activation or deactivation, occur over a broad range of timescales. Electrochemical SPMs providing detailed spatial information are generally less effective in probing fast dynamics. To overcome this limitation, one needs to develop correlative approaches combining nanoscale electrochemical mapping with time-resolved optical/spectroscopic measurements and transient SECM and SECCM methodologies capable of resolving photoelectrochemical processes on timescales from microseconds to seconds.

Overall, future progress in electrochemical photo-SPMs depends on making the measurements more quantitative, informative, and relevant to real working photocatalysts. Combined with transport and kinetic modeling, these techniques should become increasingly useful for understanding local photoelectrochemical behavior and guiding the design of better photocatalysts and photoelectrodes.

## Author contributions

All authors contributed to the first draft and approved the final version of the manuscript.

## Conflicts of interest

The authors declare that they have no conflicts of interest.

## Data Availability

No primary research results, software or code have been included and no new data were generated or analysed as part of this review.
